# Efficacy and Tolerability of Perampanel as Adjunctive Therapy in Chinese Patients With Focal-Onset Seizures: An Observational, Prospective Study

**DOI:** 10.3389/fneur.2021.731566

**Published:** 2021-08-30

**Authors:** Ranran Zhang, Shan Qiao, Xiqin Fang, Kemo Wang, Yanting Shi, Qianwen Du, Tingting Yang, Xuewu Liu

**Affiliations:** ^1^Department of Neurology, Qilu Hospital, Cheeloo College of Medicine, Shandong University, Jinan, China; ^2^Department of Neurology, The First Affiliated Hospital of Shandong First Medical University, Jinan, China; ^3^Institute of Epilepsy, Shandong University, Jinan, China

**Keywords:** epilepsy, anti-seizure medication, focal seizures, perampanel, seizure freedom

## Abstract

**Purpose:** To evaluate the efficacy and tolerability of adjunctive perampanel (PER) in Chinese patients with focal-onset seizures, with or without secondarily generalized tonic-clonic seizures.

**Methods:** Fifty-six patients aged 14–72 years were recruited consecutively in this single-center prospective observational study. All patients received PER as add-on treatment on the basis of clinical judgment. Seizure frequency, adverse events (AEs), and retention rates were obtained at 3 and 6 months after PER introduction.

**Results:** The overall response rates were 60 and 71.1% after 3 and 6 months, respectively, and the freedom of seizures at the same points were reached in 8 and 15.8%. The retention rates were 89.3% at the 3-month follow-up and 67.9% at the 6-month follow-up. The overall incidence of adverse events was 55.4%. The leading reported AEs were dizziness (39.3%) and somnolence (25%).

**Conclusions:** Our study confirmed the efficacy and tolerability of adjunctive PER in Chinese patients in real-life conditions. Based on our treatment experience, a lower maintenance dose of PER would be needed in Chinese patients.

## Introduction

Epilepsy is one of the most common chronic diseases of the central nervous system, affecting about 50 million people worldwide (1). In China, the prevalence of epilepsy is about 4.5–7.0 per 1,000, and the incidence is estimated as 28.8–35.0 per 100,000 (2). Anti-seizure medication (ASM) therapy is the main treatment for epilepsy, but about 30% of patients still do not successfully respond to conventional ASMs and develop into refractory epilepsy (3). Therefore, there is an urgent need to develop and apply newer ASMs with novel mechanisms of action.

Perampanel, one of the latest ASMs, is a highly selective and non-competitive antagonist of α-amino-3-hydroxy-5-methyl-4-isoxazolepropionic acid (AMPA) receptor (4), which is the main subtype of ionotropic glutamate receptor that mediates rapid excitatory neurotransmission in the central nervous system (CNS) and plays an important role in epileptic activity (5).

Perampanel is approved as adjunctive therapy for focal-onset seizures with or without generalization and primary generalized tonic-clonic seizures in patients' aged 12 years and older in more than 50 countries, and has been approved as monotherapy and adjunctive therapy for focal-onset seizures with or without secondarily generalized seizures in patients over 4 years of age in the United States (6). Multiple randomized, double-blind, placebo-controlled trials and real-life studies have confirmed the efficacy and safety of perampanel as adjunctive therapy for focal and generalized seizures (7–10). Furthermore, perampanel was also efficacious and tolerated in children and adolescents (11–13). In addition, the good efficacy and safety of perampanel as a monotherapy has been demonstrated (14). In September 2019, perampanel was approved for use in China as adjunctive treatment for focal-onset seizures, with or without secondarily generalized tonic-clonic seizures (sGTCS) in patients aged ≧12 years. The recent approval of perampanel means that few real-life studies on the use of perampanel in Chinese patients have been reported.

Therefore, we performed the present prospective study to evaluate the efficacy and tolerability of PER as add-on therapy in Chinese patients with focal-onset seizures, with or without secondarily generalized tonic-clonic seizures.

## Methods

### Subjects

Patients with focal-onset seizures (with or without sGTCS) who visited Qilu Hospital of Shandong University from February 2020 to September 2020 were enrolled in this study. The study was performed in accordance with the Declaration of Helsinki and was approved by the Medical Ethics Committee of Qilu Hospital of Shandong University (NO.KYLL-202008-046). All subjects and their guardians (in the case of children) gave the informed consent to participate. The inclusion criteria were (a) age ≥12 years old; (b) diagnosed as focal-onset seizures (with or without sGTCS) based on clinical presentations and EEG confirmation, according to International League Against Epilepsy classifications of seizures and epilepsy (15, 16); (c) seizures were not controlled with ≥1 ASM. The exclusion criteria: (a) psychogenic non-epileptic seizures; (b) status epilepticus; (c) primary generalized tonic-clonic seizures; (d) lactation or pregnancy; (e) alcohol or drug abuse; (f) psychiatric disease, severe cognition impairment, and severe diseases of other systems (patients with severe circulatory system diseases, hematologic system diseases, malignant tumors, and immunocompromised functions).

### Study Design

This was a 6-month, single-center, prospective study evaluating the efficacy and safety of PER. The following clinical data of the patients were collected: age, sex, etiology of epilepsy, age at onset, duration of epilepsy, previous ASMs, concomitant ASMs, seizure frequency (monthly seizure frequency during the 3 months before starting perampanel treatment), seizure types (focal or sGTCS). Enzyme-inducing ASMs (EIASMs) included carbamazepine, oxcarbazepine, phenobarbital, and phenytoin. All patients enrolled were treated with PER once daily at bedtime; the initial dose was 2 mg and titrated by 2 mg every 2 weeks depending on clinical response and tolerability, gradually increasing to a maximum tolerable dose. And there were no change in concomitant ASMs (neither changes of types of concomitant ASMs nor changes of doses of concomitant ASMs). Patients were followed up at 1, 3, and 6 months after PER add on.

### Outcome Evaluation

Efficacy was assessed by measuring changes in seizure frequency at 3 and 6 months follow up compared with baseline. The baseline was 3 months before the addition of PER and the seizure frequency was based on the patients' seizure diary. We classified patients in five categories: (1) seizure freedom, (2) reduction of seizure frequency ≧50%, (3) reduction of seizure frequency <50%, (4) no change, (5) worsened (patients with increased seizure frequency). Patients who have achieved a reduction of more than 50% or seizure free were defined as responders (the criteria for patients with sGTCS was to achieve a reduction in all seizure types).

Tolerability was evaluated by reported adverse events (AEs), PER discontinuation, and clinical laboratory tests (hematology, clinical chemistry, and urinalysis) at each follow-up. AEs were mainly based on directly reported by the patients and through specific questioning about the most known common AEs associated with PER (no standardized AE questionnaire was used).

### Statistical Analysis

For continuous variables, descriptive data were expressed as mean ± standard deviation. For categorical variables, absolute frequencies and percentages were computed. The continuous variables were assessed for normal distribution before parametric analysis. For between-group comparisons, the Student's *t*-test was used to compare continuous variables subject to normal distribution while the non-parametric Mann-Whitney *U* test was used to compare continuous variables that were not normally distributed. And categorical variables were compared using Chi-squared test or Fisher's exact test. Retention rate was calculated by counting the number of patients taking perampanel every 2 weeks by using the Kaplan-Meier survival analysis. All statistical analyses were performed using the statistical software SPSS version 21.0. The threshold of statistical significance was *p* < 0.05.

## Results

### Subjects

We enrolled 62 patients, but six of them did not receive any dose of perampanel (four patients refused to sign the informed consent, and two patients worried about adverse events of PER), so their data were not included in this study. Thus, 56 patients (29 females, 27 males) were finally recruited. The mean age was 30.1 ± 16.3 years (range 14–72 years). The baseline demographic and clinical characteristics of the patients are shown in Table 1. The etiology was classified according to the International League Against Epilepsy classification of epilepsies, including genetic (*n* = 4), structural (*n* = 22), immune (*n* = 2), metabolic (*n* = 2), infection (*n* = 6), and unknown etiology (*n* = 20). Among all patients enrolled in the study, 20 (35.7%) had focal seizures and 36 (64.3%) had sGTCS, and their monthly seizure frequency was 26.2 ± 38.8.

**Table 1:** Demographical and clinical characteristics of patients.

**Total (*n*)**	**56**
Age, years (mean ± SD)	30.1 ± 16.3
Sex, Male/Female, *n* (%)	27/29 (48.2/51.8)
Age at epilepsy onset, years (mean ± SD)	21.3 ± 17.0
Epilepsy duration, years (mean ± SD)	8.9 ± 8.8
Etiology	
Structural, *n* (%)	22 (39.3)
Genetic, *n* (%)	4 (7.1)
Immune, *n* (%)	2 (3.6)
Metabolic, *n* (%)	2 (3.6)
Infection, *n* (%)	6 (10.7)
Unknown, *n* (%)	20 (35.7)
Seizure type	
Focal, *n* (%)	20 (35.7)
Combined generalized and focal seizures, n (%)	36 (64.3)
Monthly seizure frequency (mean ± SD)	26.2 ± 38.8
Previous ASMs (mean ± SD)	2.3 ± 1.0
Concomitant ASMs (mean ± SD)	1.9 ± 0.9
Concomitant EIASMs, *n* (%)	27 (48.2)
Concomitant non-ASMs, *n* (%)	29 (51.8)

*ASMs, Anti-seizure medications; EIASMs, enzyme inducer ASMs*.

All patients were taking at least 1 ASM at the time of initiation of PER; the mean number of concomitant ASMs was 1.9 ± 0.9. The types of concomitant ASMs are shown in Table 2. Valproic acid (VPA) was the most frequent concomitant ASM (64.3%), followed by levetiracetam (LEV) (55.4%), oxcarbazepine (OXC) (26.8%), and carbamazepine (CBZ) (21.4%). Concomitant ASMs were also classified as EIASMs and non-EIASMs (any other ASMs).

**Table 2:** Anti-seizure medications combined with perampanel.

**Concomitant ASMs**	**n (%)**
VPA	36 (64.3)
LEV	31 (55.4)
OXC	15 (26.8)
CBZ	12 (21.4)
LTG	7 (12.5)
TPM	6 (10.7)
LAC	2 (3.6)
CLN	1 (1.8)

*VPA, Valproic acid; LEV, levetiracetam; OXC, oxcarbamazeine; CBZ, carbamazepine; LTG, lamotrigine; TPM, topiramate; LAC, lacosamide; CLN, clonazepam*.

### PER Dose and Retention Rate

The starting dose of perampanel is 2 mg/d before bedtime, and then increased by 2 mg/d every 2 weeks to reach the maximum tolerated dose with good seizure control. The usual target dose was 6–8 mg/d, but the adjustments of the dose were at the discretion of the physician based on the patients' clinical response.

**Figure 1:**
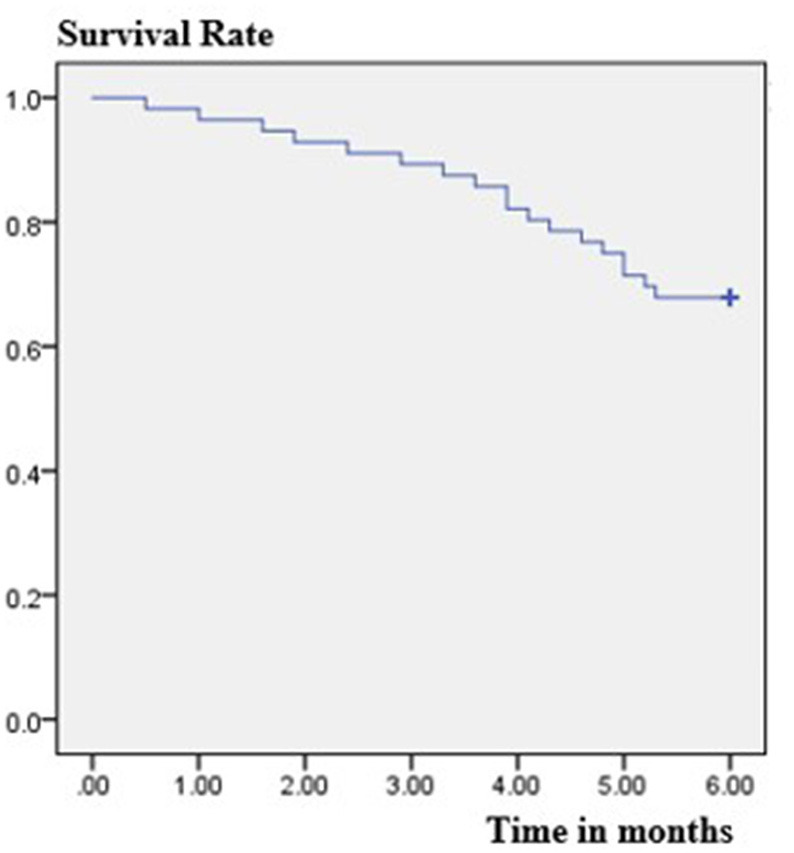
Estimated retention rate in the patients treated with adjunctive perampanel using Kaplan-Meier survival analysis.

**Figure 2:**
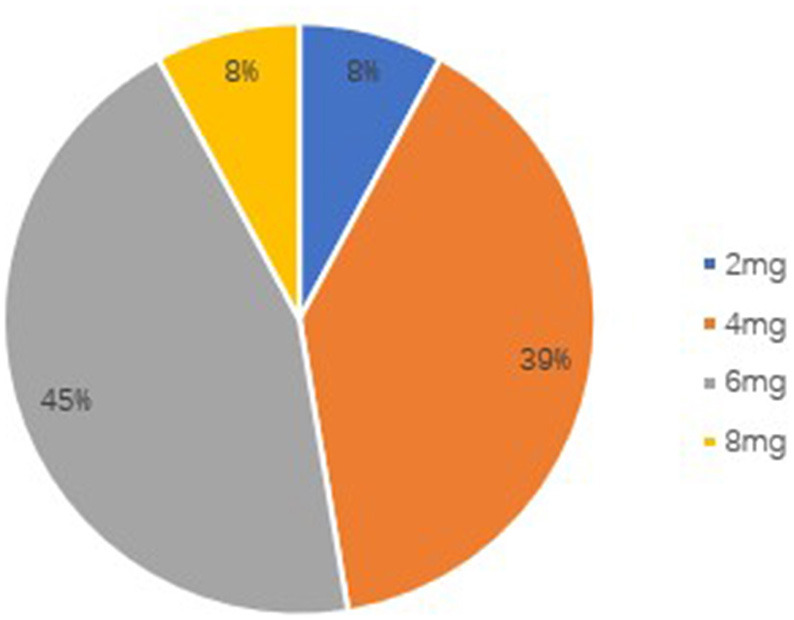
Patients' final maintenance dose of PER.

The retention rate at 3 months was 89.3% (50/56), because six patients discontinued PER. Four of them discontinued due to severe AEs, one due to worsening in seizure frequency, and one due to both events. The retention rate at 6 months was 67.9% (38/56), because four patients were lost to follow-up and eight patients discontinued PER. Of these eight patients, due to AEs occurrence in five subjects, no change of seizure frequency in two patients and worsening of seizure frequency in one patient. The overall retention rate was shown in Figure 1. At the 6-month follow-up, the patients' final maintenance dose was recorded (Figure 2). The most frequent maintenance dose was 6 mg, taken by 17 (44.7%) patients. Also, 4 mg was taken by 15 (39.5%) patients, 2 mg was taken by 3 (7.9%) patients, and 8 mg was taken by 3 (7.9%) patients. Subjects taking EIASMs with PER did have higher maintenance dose compared with those taking non-EIASMs (5.3 vs. 4.5 mg).

### Efficacy

**Figure 3:**
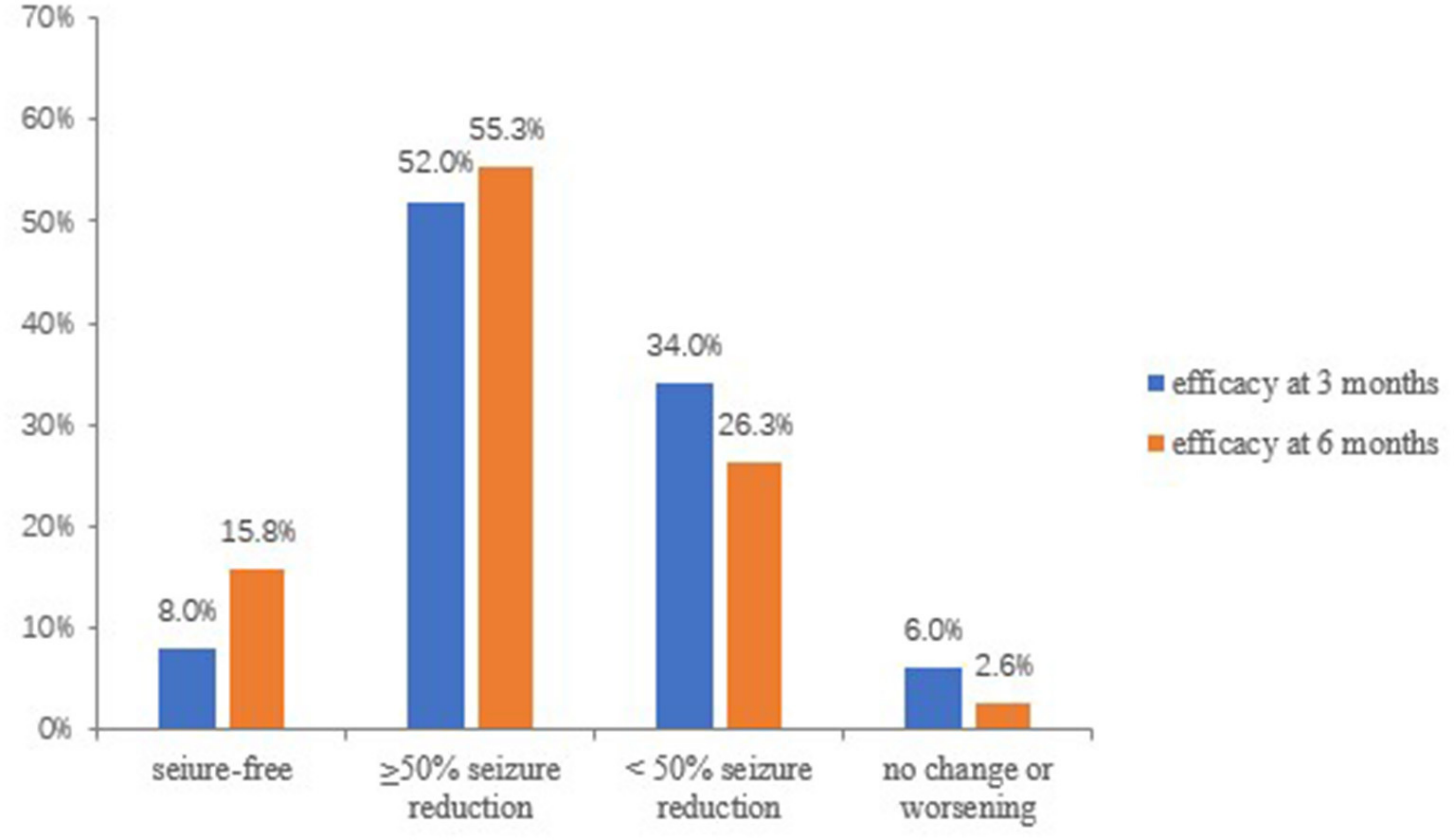
Efficacy of PER at 3 and 6 months follow-up.

At 3 months, 50 patients were available for efficacy evaluations. The overall response rate (≥50% seizure reduction) at 3 months was 60% (30/50). In details, seizure freedom was achieved in 8% (4/50) of the patients, 52.0% (26/50) had a reduction ≥50%, 17 (34.0%) had a reduction <50%, 2(4.0%) showed no change in seizure frequency, and 1 (2.0%) out of 50 patients experienced a worsening in seizure frequency (Figure 3).

Thirty-eight patients were available for efficacy evaluations at the 6 months follow-up. The overall response rate at 6 months increased to 71.1% (27/38), and 15.8% (6/38) became seizure-free, 55.3% (21/38) had a reduction ≥50% of seizure frequency. Ten (26.3%) had a reduction <50%, and 1 (2.6%) showed no change in seizure frequency. In addition, none of the patients experienced a worsening of seizure frequency (Figure 3).

After the last follow-up, patients were divided into responders and non-responders. A comparison of their demographics and related variables was shown in Table 3. There were no significant differences in age, gender, age of onset, between responders and non-responders. The duration of epilepsy in non-responders was longer than that in responders (*p* = 0.038). Interestingly, as to etiology, patients with structural (16/56, 28.6%) and unknown etiology (15/56, 26.7%) seemed to have better responses although this result had weak statistical significance.

**Table 3:** Comparison of demographics and related variables between responders and non-responders.

	**Responders**	**Non-responders**	***P***
Age, years (mean ± SD)	30.4 ± 16.9	29.3 ± 15.3	1.000
Sex, Male/Female, *n* (%)	22/18 (39.3/32.1)	5/11 (9.0/19.6)	0.108
Age at epilepsy onset, years (mean ± SD)	22.2 ± 18.6	19.3 ± 12.5	0.964
Epilepsy duration, years (mean ± SD)	7.7 ± 7.1	11.6 ± 9.3	0.038
Etiology			
Structural, *n* (%)	16 (28.6)	6 (10.7)	0.863
Genetic, *n* (%)	2 (3.6)	2 (3.6)	0.325
Immune, *n* (%)	1 (1.8)	1 (1.8)	0.495
Metabolic, *n* (%)	1 (1.8)	1 (1.8)	0.495
Infection, *n* (%)	5 (8.9)	1 (1.8)	0.371
Unknown, *n* (%)	15 (26.7)	5 (8.9)	0.659
Seizure type			0.202
Focal, *n* (%)	11 (19.4)	9 (16.1)	
Combined generalized and focal seizures, *n* (%)	29 (51.8)	7 (12.7)	
Monthly seizure frequency (mean ± SD)	18.9 ± 29.4	45.3 ± 51.9	0.023
Previous ASMs	2.2 ± 0.8	2.7 ± 0.8	0.045
Mean number of concomitant ASMs	2.1 ± 0.5	2.5 ± 0.8	0.047
Concomitant EIASM(s)			0.866
Concomitant EIASMs	19 (33.9)	8 (14.3)	
Concomitant non-EIASMs	21 (37.5)	8 (14.3)	
Concomitant ASM(s)			
VPA	29 (51.8)	7 (12.5)	0.043
LEV	26 (46.4)	5 (8.9)	0.022
OXC	10 (17.8)	5 (8.9)	0.633
CBZ	9 (16.1)	3 (5.4)	0.757
LTG	4 (7.1)	3 (5.4)	0.395
TPM	4 (7.1)	2 (3.6)	1.000
LAC	1 (1.8)	1 (1.8)	0.494
CLN	1 (1.8)	0 (0.0)	–

*ASMs, Anti-seizure medications; EIASMs, enzyme inducer ASMs; VPA, Valproic acid; LEV, levetiracetam; OXC, oxcarbamazeine; CBZ, carbamazepine; LTG, lamotrigine; TPM, topiramate; LAC, lacosamide; CLN, clonazepam*.

Compared with subjects with focal-onset seizures, patients with sGTCS accounted for a higher percentage of responders (51.8%, 29/56), although without statistical significance (*p* = 0.202). And the baseline seizure frequency of non-responders is higher than that of responders (*p* = 0.023). Moreover, responder and non-responder patients significantly differed for number of previous ASMs (*p* = 0.045) and number of concomitant ASMs (*p* = 0.047). The number of previous ASMs and concomitant ASMs were significantly smaller in responders than in non-responders.

There were no significantly statistical differences in response rates between subjects taking EIASMs (33.9%, 19/56) with PER and those taking non-EIASMs (37.5%, 21/56) (*p* = 0.866). In concomitant ASMs, VPA, and LEV were more frequently co-administrated in responder than non-responder patients (*p* = 0.043, *p* = 0.022, respectively). Moreover, the combined application of VPA, LEV, and PER was the highest proportion of responders (33.3%, 9/27), followed by the combination of LEV and PER (18.5%, 5/27) (Table 4).

**Table 4:** ASMs combination regimen in responders.

**ASMs combination regimen**	***n* (%)**
VPA, LEV, PER	9 (33.3)
LEV, PER	5 (18.5)
VPA, OXC, PER	2 (7.4)
VPA, CBZ, PER	2 (7.4)
VPA, LEV, CBZ, LTG	2 (7.4)
VPA, PER	1 (3.7)
VPA, CBZ, PER	1 (3.7)
VPA, LTG, PER	1 (3.7)
VPA, OXC, PER	1 (3.7)
LEV, OXC, PER	1 (3.7)
LEV, CLN, PER	1 (3.7)
OXC, PER	1 (3.7)

*ASMs, Anti-seizure medications; VPA, Valproic acid; LEV, levetiracetam; OXC, oxcarbamazeine; CBZ, carbamazepine; LTG, lamotrigine; TPM, topiramate; LAC, lacosamide; CLN, clonazepam*.

### Safety

**Figure 4:**
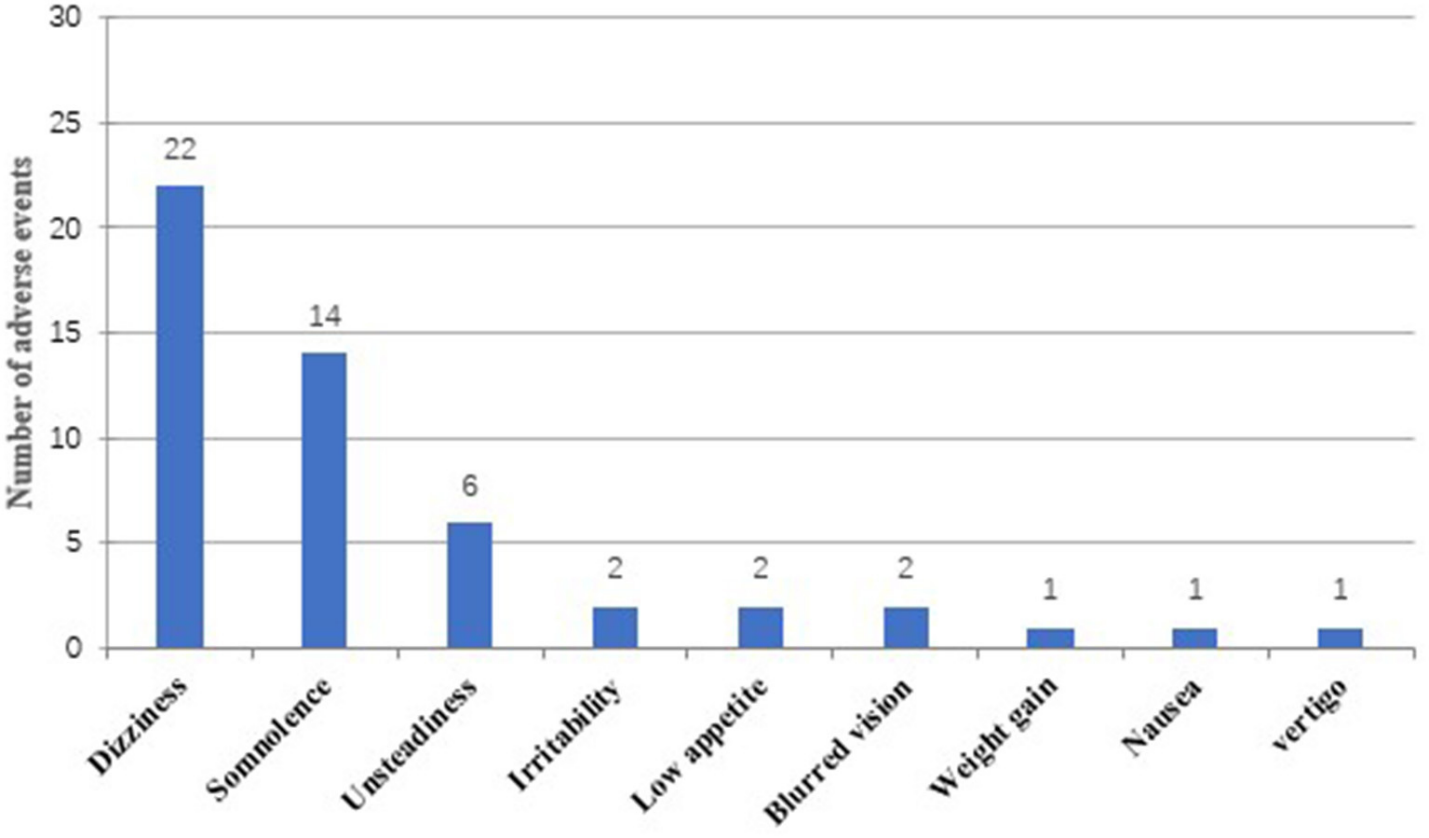
Adverse events associated with PER treatment.

Overall, 31 out of 56 patients (55.4%) experienced at least one AE during treatment with PER (6 months follow-up), and a total of 51 AEs were reported (Figure 4). There was no significant difference in the mean dose of PER between patients experiencing AEs and those without (5.0 ± 1.5 vs. 4.8 ± 1.4 mg; *p* = 0.907). Dizziness was the most common adverse event (39.3%, 22/56), followed by somnolence (25%, 14/56) and unsteadiness (10.7%, 6/56). Irritability (3.6%, 2/56), blurred vision (3.6%, 2/56), low appetite (3.6%, 2/56), weight gain (1.8%, 1/56), nausea (1.8%, 1/56), and vertigo (1.8%, 1/56) were less frequently mentioned. In general, AEs were mild to moderate and could be tolerated by most patients. Discontinuation for AEs occurred in 10 patients (17.9%) (some with more than one AE). Specifically, six patients withdrew from PER treatment for dizziness, three for unsteadiness, two for blurred vision, and one for low appetite.

Some laboratory test changes were also observed, but most were within the normal range and had no clinical significance.

## Discussion

This observational study evaluated the efficacy and safety of PER as an add-on therapy for the treatment of Chinese patients with focal-onset seizures, with or without sGTCS. In our population, we found a responder rate of 60% after 3 months and 71.1% after 6 months of PER administration, with 8% and 15.8% of patients achieving seizure freedom at 3 and 6 months, respectively. The percentage of patients who were responders and seizure free in the current study was slightly higher than that reported in other real-life studies (17–19). The slightly higher responder and seizure-free rates may be due to the differences in patient populations between the studies (e.g., duration of epilepsy, seizure types, and previous treatments). Moreover, the majority of the subjects included in this study were patients with less refractory epilepsy. Interestingly, our study found that patients with structural and unknown etiology seemed to have a better response to PER. The percentage of responders in patients with sGTCS was higher than in patients without sGTCS (51.8 vs. 19.4%), and this result was similar to other studies (9, 20, 21); after following long-term PER treatment, improvements were particularly notable for sGTCS. The efficacy of PER against generalized seizure types may be related to its mechanism of action as a selective AMPA receptor antagonist (22).

Likewise to previous reports (9, 17, 23), we found that the number of previous and concomitant ASMs may be negatively correlated with efficacy of adjunctive PER. The higher the number of ASMs, the worse the response to treatment. Moreover, in our study, the baseline seizure frequency of non-responders was higher than that of responders. Therefore, these results might indicate that patients who failed to respond to PER addition had more refractory epilepsy.

Since enzyme inducers may interfere with the metabolism of perampanel and reduce serum drug concentrations, our study confirmed the influence of EIASMs on PER efficacy. Unlike what other studies have shown (17, 24, 25), although subjects taking EIASMs with PER did have higher maintenance dose compared with those taking non-EIASMs, the effect of EIASMs on PER efficacy was not reduced. This difference may be due to demographic characteristics and pharmacokinetic characteristics of different races. Therefore, the current study suggested that the effect of enzyme inducers on PER may be ambiguous and some patients receiving concomitant EIASMs might have a good response, and physicians should be aware that some patients who are also treated with EIASMs may experience low serum concentrations of PER and need to increase the dosage of PER.

In the current study, the 3-month retention rate was 89.3% and the 6-month retention rate is 67.9%, which was similar to the results in FYDATA study, where the 3-month retention rate is 88.4% and the 6-month retention rate is 74.4% (19). And another study in South Korea documented a 6-month retention rate of 71.3% (26). Therefore, similar retention rates among the studies may suggest that the tolerability was similar. In addition, the higher retention rate of patients in our study may be related to the good response rate, and on the other hand, the once-daily, night-time dosing regimen of PER may help improve medication adherence in patients with epilepsy (27).

One interesting finding is that the mean maintenance dose of perampanel in our study (5.1 ± 1.5 mg) was lower compared to other studies (17, 27, 28). One multicenter study from Europe reported that 6 mg and 8 mg were the main maintenance doses and another *post-hoc* analysis showed that the main maintenance doses of 8 and 12 mg could have good efficacy, however, patients taking doses of 4 and 6 mg (39.5, 44.7%, respectively) accounted for the majority of patients in the current study. Moreover, a *post-hoc* analysis of four phase III studies confirmed the efficacy and safety of adjunctive perampanel 4 mg/d in the treatment of focal epilepsy (29). Consistent with previous studies, the mean maintenance dose of perampanel in South Korea study was 4.39 ± 1.97 mg (26). Furthermore, in an Italian multicenter long-term follow-up study, Operto et al. reported that perampanel was effective in association with 1 or 2 ASMs in both pediatric and adult patients, without having to use a high dose of the drug (10). Thus, these results might suggest that a lower maintenance dose of PER is needed in some patients.

Similar to other studies, among the concomitant ASMs, the majority of patients took LEV and VPA (26, 30). In the present study, VPA was the most common concomitant ASM (64.3%), followed by LEV (55.4%). Interestingly, we analyzed the effect of concomitant ASMs on response rates and found that patients with combined application of LEV or VPA had better responses. What's more, among the ASMs combination regimens for responders, the triple combination of VPA, LEV, and PER (33.3%) was the most frequent regimen, followed by the combination of LEV and PER (18.5%). Therefore, the prospect of PER combined with other ASMs could be optimistic and could provide patients with a better treatment option. In our study, VPA, LEV, and PER were commonly used and effective combination regimens.

Nearly half of patients experienced adverse events; dizziness and somnolence were the most frequent reported AEs after taking PER, which were similar to what has been reported in previous literatures (8, 18, 19, 31). Interestingly, unlike other studies (9, 17), the incidence of AEs related to psychiatric symptoms (irritability, depression, aggressive behavior) was low in the population of our study. These results may be explained by the differences in baseline characteristics of patients and physiological uniqueness between the ethnic groups. Most of the AEs were mild to moderate and consistent with other reports (9, 19, 28, 32); there did not seem to be a clear correlation between dosage of PER and the probability of AEs. Of the 10 patients who stopped taking PER, dizziness was also the most common AE leading to withdrawal, following by unsteadiness, blurred vision, and low appetite. However, these AEs subsided with dosage reduction and withdrawal without causing serious diseases. Moreover, it is worth mentioning that the occurrence of AEs cannot be attributed to a single addition of a new drug, but may also be caused by different combinations and the total drug load (18).

There are several limitations in our study. First, this was a single-center short-term observational study that included only a limited number of patients. Therefore, randomized double-blind clinical trials on a larger number of patients with longer follow-up are necessary in the future. Second, serum levels of PER were not measured, and we could not precisely assess the influence of enzyme inducers on plasma levels associated with seizure control and AEs. Finally, as we did not use a standardized questionnaire for assessing AEs, the incidence of adverse events caused by PER treatment may be inaccurate.

## Conclusion

In conclusion, as the first prospective real-life study in Chinese patients, our findings confirmed the good efficacy and tolerability of adjunctive PER in patients with focal-onset seizures, with or without combined generalized and focal seizures. The concomitant use of EIASMs did not appear to reduce the efficacy of PER in seizure control. In terms of maintenance dose, a lower maintenance dose of perampanel would be needed in Chinese patients. The combined application of PER and other ASMs may provide a better treatment option for patients; VPA and LEV were commonly used and effective combination ASMs.

## Data Availability Statement

The original contributions generated for the study are included in the article/supplementary material, further inquiries can be directed to the corresponding author/s.

## Ethics Statement

The studies involving human participants were reviewed and approved by Medical Ethics Committee of Qilu Hospital of Shandong University. Written informed consent to participate in this study was provided by the participants' legal guardian/next of kin. Written informed consent was obtained from the individual(s), and minor(s) legal guardian/next of kin, for the publication of any potentially identifiable images or data included in this article.

## Author Contributions

RZ wrote the manuscript. XL and SQ contributed to the conception of the study. XF and KW helped perform the statistical analysis. YS and QD helped collected the clinical data. TY performed the follow-up.

## Conflict of Interest

The authors declare that the research was conducted in the absence of any commercial or financial relationships that could be construed as a potential conflict of interest.

## Publisher's Note

All claims expressed in this article are solely those of the authors and do not necessarily represent those of their affiliated organizations, or those of the publisher, the editors and the reviewers. Any product that may be evaluated in this article, or claim that may be made by its manufacturer, is not guaranteed or endorsed by the publisher.
